# A Home-Based Interdisciplinary Intervention to Enhance Functionality in Oncology Patients: Results from a Clinical Trial

**DOI:** 10.3390/jcm14134417

**Published:** 2025-06-20

**Authors:** Eduardo José Fernández-Rodríguez, Celia Sánchez-Gómez, Maria Isabel Rihuete-Galve, Emilio Fonseca-Sánchez, Juan Jesús Cruz-Hernández

**Affiliations:** 1 Department of Nursing and Physiotherapy, Universidad de Salamanca, 37008 Salamanca, Spain; rihuete@usal.es; 2 Institute of Biomedical Research of Salamanca (IBSAL), 37007 Salamanca, Spain; efonseca@usal.es (E.F.-S.); jjcruz@usal.es (J.J.C.-H.); 3 Department of Developmental and Educational Psychology, University of Salamanca, 37007 Salamanca, Spain; 4 Department of Medical Oncology, University Hospital of Salamanca, 37007 Salamanca, Spain; 5 Department of Medicine, University of Salamanca, 37007 Salamanca, Spain

**Keywords:** cancer rehabilitation, dyspnoea, oncology patients, quality of life

## Abstract

**Background/Objectives:** Dyspnoea and functional decline are common among cancer patients with associated respiratory conditions. This study aimed to evaluate the effectiveness of an Effort Re-education Programme (ERP) in improving functionality and quality of life in hospitalised oncology patients compared to Conventional Clinical Practice (CCP). **Methods:** A stratified, randomised, prospective clinical trial was conducted involving 65 patients with cancer and associated respiratory conditions. Participants were assigned to either a control group (CCP) or an experimental group (ERP + CCP). Functionality (Barthel Index), health-related quality of life (EORTC QLQ-C30), overall performance (Karnofsky Scale), and instrumental activities of daily living (Lawton and Brody Scale) were assessed at baseline and one month post-discharge. **Results:** The ERP group showed significantly greater improvements in all outcome measures: Barthel Index (mean change: +18.33 vs. +6.19), EORTC QLQ-C30 (+16.4 vs. +6.6), Karnofsky (+18.75 vs. +5.6), and Lawton–Brody (+2.78 vs. +0.78), all with *p* < 0.001 and moderate-to-large effect sizes (Cohen’s d = 0.72–1.19). No readmissions were reported in the ERP group, versus 37.5% in the control group. **Conclusions:** The ERP significantly improves basic and instrumental functionality, autonomy, and health-related quality of life in oncology patients with respiratory conditions. These findings support the integration of Functional Re-education Programmes into routine clinical practice as a complement to standard care.

## 1. Introduction

Advances in early diagnosis and oncological treatments have significantly increased survival rates among cancer patients, giving rise to the concept of “long-term survivors” [1].

However, this prolonged survival also entails greater exposure to successive lines of therapy, which, while improving prognosis, generate significant side effects that negatively impact patients’ functionality and quality of life. Among the most notable effects are cancer-related fatigue, dyspnoea, and emotional disturbances such as anxiety and depression [2,3].

In particular, dyspnoea is a highly prevalent symptom among patients with associated respiratory conditions and is especially debilitating in advanced stages of the disease. It is estimated that up to 73% of patients with lung cancer experience dyspnoea, and this figure rises to 41% among those receiving palliative care, with nearly half perceiving it as moderate to severe [4,5].

Many patients develop patterns of physical effort avoidance due to fear of exacerbating their breathlessness, which further contributes to functional decline [6].

This fear–avoidance behaviour has also been described in other chronic conditions, such as persistent pain, chronic fatigue syndrome, and fibromyalgia [7,8].

In oncology, this pattern contributes to the development of the so-called “respiratory patient cycle”, in which decreased activity leads to deterioration in physical condition, which in turn exacerbates exertional dyspnoea and increases functional dependence [9].

Traditionally, the treatment of dyspnoea in these patients has been primarily pharmacological. However, due to its multifactorial aetiology, this approach is often insufficient to achieve adequate symptom control. It is, therefore, necessary to incorporate functional interventions that promote re-adaptation to daily life from a holistic perspective.

In this regard, clinical practice guidelines such as those from the NCCN recommend complementing pharmacological management with non-pharmacological strategies, including health education and energy conservation techniques, integrated into functional rehabilitation programmes [10].

Various studies have demonstrated the effectiveness of such programmes in improving both quality of life and functionality. A meta-analysis including over 11,000 patients showed a significant improvement following the implementation of non-pharmacological interventions (physical exercise, resistance exercise, mindfulness, multicomponent interventions) (effect size: 0.30; 95% CI: 0.25–0.36; *p* < 0.001) [11].

Moreover, other studies highlight the importance of an interdisciplinary approach and structured follow-up to ensure adherence and the success of these interventions [12,13,14,15,16,17].

In this context, multimodal intervention programmes combining tailored physical exercise, health education, and re-training in activities of daily living have shown particularly promising results in cancer patients. These interventions must be adapted to each patient’s functional and clinical status, with priority given to assessing both basic and instrumental functionality, overall quality of life, and sociodemographic factors that may influence clinical progress.

Therefore, the implementation of a supervised Functional Re-education Programme is proposed, aimed at patients with cancer and associated respiratory conditions who have recently been discharged from the hospital. This programme was delivered in the patient’s home and was overseen by an interdisciplinary team comprising occupational therapists, nursing professionals, and medical specialists. The intervention aims to complement Conventional Clinical Practice through a strategy focused on improving patients’ functionality, autonomy, and quality of life.

### 1.1. Primary Objective

The primary objective of this study is to evaluate the effectiveness of an Effort Re-education Programme (ERP), compared to Conventional Clinical Practice (CCP), in improving functionality in basic activities of daily living in hospitalised cancer patients with associated respiratory conditions, as measured by the Barthel Index.

### 1.2. Secondary Objectives

The secondary objectives of this study are as follows:

1. To analyse the impact of the Effort Re-education Programme on health-related quality of life, assessed using the EORTC QLQ-C30 questionnaire.
2. To evaluate the progression of patients’ general functional status throughout this study, using the Karnofsky Performance Scale.
3. To determine the effects of the programme on the ability to carry out instrumental activities of daily living, measured using the Lawton and Brody Scale.
4. To explore correlations between functionality, quality of life, general functional status, and level of autonomy at baseline, in order to identify potential clinical and sociodemographic factors associated with clinical outcomes.

## 2. Materials and Methods

### 2.1. Study Design and Setting

This is a randomised, stratified, prospective, longitudinal clinical trial with a fixed parallel-group allocation design, comprising an experimental group and a control group. This study took place in a mixed setting: within the specialist care context of the University Hospital Complex of Salamanca (CAUSA), specifically in the Department of Medical Oncology, and at the University of Salamanca, in the Teaching and Clinical Unit of Occupational Therapy (UDATO).

### 2.2. Participants and Sample

#### 2.2.1. Target Population

The target population comprised cancer patients with associated respiratory conditions who were hospitalised at the time of inclusion. Participants were selected through consecutive sampling according to the following criteria:

#### 2.2.2. Eligibility Criteria

*Inclusion Criteria:*

The inclusion criteria for this study were as follows:

- Any histopathological diagnosis of newly diagnosed or relapsed cancer as the reason for hospital admission.
- Hospitalisation in the Oncology Department of the University Hospital of Salamanca.
- Moderate to severe dependency: Barthel Index score between 20 and 55 points.
- Signed informed consent authorising voluntary participation.

*Exclusion Criteria:*

The exclusion criteria for this study were as follows:

- Cognitive impairment, defined as a score <24 on the Mini-Mental State Examination (MMSE).
- Patients with a history of lymphoma or severe cardiac involvement.

*Withdrawal Criteria:*

The withdrawal criteria for this study were as follows:

- Death of the patient.
- Disease progression leading to a terminal condition.
- Hospitalisation at the time of home follow-up.
- Incomplete final assessment.

### 2.3. Sample Size Calculation

The sample size was calculated based on this study’s primary variable, the Barthel Index, considering a minimally clinically important difference of 4.8 points. This estimate was derived from a pilot study conducted in a similar cancer population, which observed a standard deviation of 9.7 points. The pilot study was conducted in the 4 months prior to the start of the study described above.

To detect this difference with a statistical power of 80% and a two-tailed significance level of α = 0.05, it was determined that 32 participants per group were required. Assuming a 10% attrition rate during follow-up, the sample size was increased accordingly. The calculation was performed using the Epidat 4.2 software.

### 2.4. Randomisation and Blinding

Participants meeting the inclusion criteria were randomly assigned to the intervention group (IG) or control group (CG), according to the order of baseline assessment. Randomisation was carried out by an independent researcher using Epidat 4.2, with a 1:1 allocation ratio. Allocation sequences remained concealed until the moment of assignment.

Blinding was ensured at multiple levels. Allocation and sequencing were managed by personnel not involved in the intervention or evaluation. Participants were unaware of their group allocation and the specific intervention they received. To minimise bias, evaluations were conducted by trained external researchers blinded to group assignment. Additionally, data analysts remained blinded to group allocation to ensure scientific rigour.

### 2.5. Procedures and Data Collection

#### 2.5.1. Assessment Schedule

Assessments were conducted at three time points:

1. Baseline assessment at hospital discharge (after inclusion and before randomisation).
2. Follow-up assessment at 15 days.
3. Final assessment one month after the baseline assessment.

**Figure 1:**
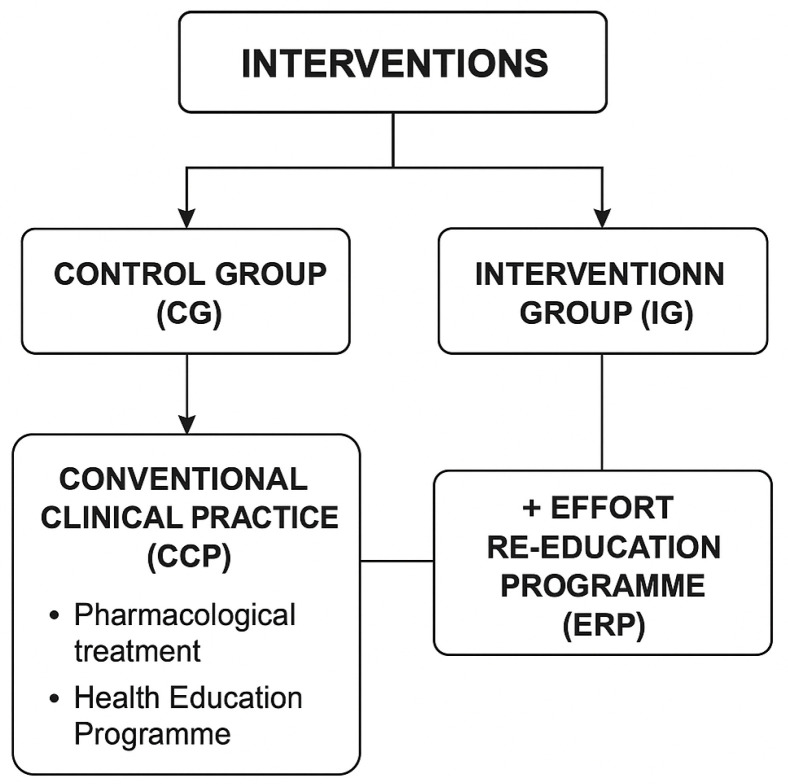
Interventions of the different study groups.

The same tools were used at each time point. Baseline data included demographic, clinical, and outcome variables. Assessments were performed by trained research personnel. The study flowchart is shown in Figure 1.

#### 2.5.2. Reporting of Results

Participants may request an individualised report containing the results of their assessments.

### 2.6. Variables

#### 2.6.1. Primary Variable

*Functionality in basic activities of daily living:* This metric was assessed using the Barthel Index [18], validated in Spanish-speaking populations. The scores range from 0 (total dependence) to 100 (complete independence).

**Secondary Variables**

- *Health-related quality of life (HRQoL):* This metric was measured using the EORTC QLQ-C30 questionnaire [19], which assesses multiple functional dimensions and cancer-related symptoms.
- *Overall quality of life/functional status:* This metric was assessed using the Karnofsky Performance Scale [20], with scores ranging from 0 (death) to 100 (normal activity, no evidence of disease).
- *Instrumental activities of daily living (IADLs):* This metric was assessed using the Lawton and Brody Scale [21], which rates the level of independence in tasks such as using the telephone, preparing meals, or managing finances.

**Interventions**

Two parallel intervention programmes were designed for the study groups. The first group, the control group (CG), received Conventional Clinical Practice (CCP), which included pharmacological treatment and a Health Education Programme. The second group, the Intervention Group (IG), received the control group intervention plus an Effort Re-education Programme (ERP). This is more clearly detailed in Figure 1. Interventions of the different study groups.

Both programmes were structured and supervised by the research team at the University of Salamanca (Spain).

**A. Control Group (CG): Conventional Clinical Practice—Pharmacological Treatment + Health Education Programme**

Participants received a Health Education Programme in the form of a booklet provided upon discharge, including information on the importance of physical activity, nutrition, and hydration in maintaining a healthy lifestyle.

**B. Intervention Group (IG): Effort Re-education Programme to Improve Performance in Activities of Daily Living**

This consisted of the following components:

1. **Functional Re-education**

Initiated prior to hospital discharge and continued at home, including:

- Direct intervention in activities of daily living (ADLs).
- Energy conservation techniques (ECTs).
- Sleep hygiene recommendations based on NCCN guidelines.

2. **Prescription of assistive products and environmental adaptations**

The assessment was conducted before discharge and at home to recommend assistive devices and identify environmental barriers to independence.

The intervention began upon hospital discharge and continued at home for one month.

#### 2.6.2. Visit Schedule

Each participant attended three assessment visits:

- **Baseline visit:** conducted before discharge and included full data collection and randomisation.
- **Follow-up visits (15 days and 1 month):** conducted at UDATO and followed the same structure as the baseline visit, except for sociodemographic data.

Each visit lasted approximately one hour.

#### 2.6.3. Data Analysis

The statistical analysis plan was defined prior to study initiation, with only minor adjustments made afterwards. Initially, a thorough data review and cleaning process was conducted to identify any errors in data collection, ensuring the proper application of exclusion criteria.

The analysis followed the intention-to-treat principle. The Kolmogorov–Smirnov test (*p* < 0.05) was used to assess the normality of distribution, supported by graphical histogram representations. A descriptive analysis of all study variables was then performed. Quantitative variables were reported as median and interquartile range, while qualitative variables were expressed as absolute frequencies and percentages.

For inferential analysis, the non-parametric Wilcoxon test was used to compare means between groups. The effect size (ES) of the experimental treatment was estimated using Cohen’s *d*, with 0.2 considered small, 0.5 moderate, and 0.8 or higher as large. Associations between clinical and functional variables, as well as questionnaire-derived measures, were analysed using Spearman’s rank correlation coefficient.

A 95% confidence level was used, with *p*-values < 0.05 considered statistically significant. All analyses were performed using IBM SPSS Statistics, version 28.0.1.

#### 2.6.4. Trial Registration and Scientific Rigor

This study adheres to the SPIRIT 2013 guidelines for protocol development and the CONSORT 2010 guidelines for reporting randomised clinical trials.

Trial registration: ClinicalTrials.gov (ID: NCT06035263).

## 3. Results

### 3.1. Baseline Sociodemographic and Clinical Characteristics

A total of 65 patients were included in the analysis, with 32 assigned to the control group and 33 to the experimental group. The distribution of participants, as well as the inclusion of participants, can be seen in more detail in Supplementary Material S1: CONSORT flow diagram of participants included in the trial.

The mean age was comparable between groups, at 64.59 years (SD = 14.95) in the control group and 66.24 years (SD = 12.15) in the experimental group. Regarding sex distribution, men predominated in the control group (59.4%) compared with 40.6% women; a similar proportion was observed in the experimental group, with 63.6% men and 36.4% women.

In terms of histopathological diagnosis, lung cancer was the most frequent in both groups, accounting for 43.8% of cases in the control group and 45.5% in the experimental group. Other common tumours included breast cancer (9.4% in the control group vs. 21.2% in the experimental group), colorectal cancer (9.4% in the control group vs. 6.1% in the experimental group), and prostate cancer (9.4% in the control group vs. 3.0% in the experimental group).

The proportion of patients with metastases was slightly higher in the control group (81.3%) compared with the experimental group (75.0%). Regarding hospital readmissions, 37.5% of patients in the control group experienced at least one readmission during follow-up, while no readmissions were recorded in the experimental group.

The average number of treatment lines received was 3.00 (SD = 0.92) in the control group and 3.21 (SD = 0.96) in the experimental group.

These data are summarised in Table 1.

### 3.2. Intragroup Progression

Regarding functionality as measured by the Barthel Index, a progressive improvement was observed in both groups, with a more marked increase in the experimental group. In this group, the mean score rose from 58.12 (SD = 19.78) to 76.45 (SD = 17.62), while in the control group, it increased from 55.03 (SD = 21.34) to 61.22 (SD = 20.08).

Health-related quality of life, assessed using the EORTC QLQ-C30 questionnaire, improved in both groups, with a greater magnitude of change in the experimental group. The global score rose from 58.40 (SD = 14.25) to 74.80 (SD = 13.71) in the experimental group, compared to an increase from 59.90 (SD = 13.95) to 66.50 (SD = 14.02) in the control group.

With regard to the general quality of life, measured by the Karnofsky Performance Scale, a significant improvement was also observed in both groups. In the experimental group, the mean increased from 60.00 (SD = 10.23) to 78.75 (SD = 9.87), whereas in the control group, it rose from 62.50 (SD = 11.12) to 68.10 (SD = 10.55).

Instrumental activities of daily living, measured using the Lawton and Brody Scale, showed a significant improvement in the experimental group, with mean scores increasing from 3.10 (SD = 1.48) to 5.88 (SD = 1.60), while the increase in the control group was more modest, from 3.32 (SD = 1.52) to 4.10 (SD = 1.67).

The mean number of hospitalisation days was lower in the experimental group (10.88 days; SD = 2.97) compared to the control group (12.14 days; SD = 3.26). These findings are presented in Table 1 and Table 2.

### 3.3. Between-Group Comparative Analysis

A paired samples test was used to compare outcomes between the experimental and control groups in key clinical and functional variables. The analyses revealed statistically significant differences in all variables studied, favouring the experimental group.

Regarding overall functional status, measured using the Karnofsky Scale, a significant improvement was observed in the experimental group compared with the control (t(31) = 6.72; *p* < 0.001), with a mean difference of 10.65 points (95% CI: 7.35–13.94). The effect size was large (Cohen’s *d* = 1.19; Hedges’ *g* = 1.16).

Functionality in basic activities of daily living, measured by the Barthel Index, improved significantly in the experimental group, with a mean difference of 13.45 points (95% CI: 6.89–20.01; t(31) = 4.12; *p* < 0.001). The effect size was moderate (Cohen’s *d* = 0.73; Hedges’ *g* = 0.70).

Regarding health-related quality of life (EORTC QLQ-C30), the experimental group demonstrated significantly greater improvement than the control group, with a mean difference of 8.45 points (95% CI: 4.20–12.70; t(31) = 4.09; *p* < 0.001) and a moderate effect size (Cohen’s *d* = 0.72; Hedges’ *g* = 0.69).

In terms of instrumental activities of daily living (Lawton–Brody Scale), significant improvements were also observed in the experimental group compared to the control group (mean difference = 1.45; 95% CI: 0.85–2.06; t(31) = 4.98; *p* < 0.001), with a large effect size (Cohen’s *d* = 0.88; Hedges’ *g* = 0.86).

### 3.4. Correlation Analysis

Pearson correlation analysis was conducted between the baseline clinical and functional variables. The most relevant associations are described below.

A significant positive correlation was observed between the Barthel Index and the Karnofsky Scale (r = 0.623; *p* < 0.001), indicating that higher functional autonomy is associated with better perceived general health status.

The EORTC QLQ-C30 questionnaire also showed significant positive correlations with the Barthel Index (r = 0.512; *p* < 0.001) and the Karnofsky Scale (r = 0.598; *p* < 0.001), as well as a positive correlation with the Lawton–Brody Scale (r = 0.445; *p* = 0.003), suggesting that better quality of life is related to greater functionality at all levels.

The Lawton–Brody Scale showed a positive correlation with both the Barthel Index (r = 0.488; *p* < 0.001) and the Karnofsky Scale (r = 0.461; *p* = 0.002), reflecting that the ability to perform instrumental activities is related to better overall functional status.

On the other hand, age showed a negative correlation with Barthel Index scores (r = −0.312; *p* = 0.010) and with the Lawton–Brody Scale (r = −0.350; *p* = 0.005), suggesting a decline in function associated with ageing. Age also negatively correlated with Karnofsky scores (r = −0.296; *p* = 0.015), although no significant correlation was found between age and the EORTC QLQ-C30 score (r = −0.170; *p* = 0.185).

The number of hospitalisation days was negatively correlated with the Barthel Index (r = −0.291; *p* = 0.017) and with the Karnofsky Scale (r = −0.339; *p* = 0.007), suggesting that poorer functionality is associated with longer hospital stays.

## 4. Discussion

The results of this randomised clinical trial show that the implementation of an Effort Re-education Programme (ERP) in hospitalised oncology patients with associated respiratory conditions significantly improves functionality in both basic and instrumental activities of daily living, as well as overall and health-related quality of life, compared to Conventional Clinical Practice (CCP).

In line with previous studies, such as that by Fernández et al. [22], the efficacy of comprehensive rehabilitation programmes in improving functional autonomy and reducing dyspnoea in oncology patients is confirmed. Our study builds upon these findings by demonstrating significant benefits not only in functionality (Barthel Index) but also in quality of life (EORTC QLQ-C30) and overall performance (Karnofsky Scale), with moderate-to-large effect sizes across all assessed variables.

Furthermore, the present study supports the conclusions of the meta-analysis conducted by Liu et al. [23], which demonstrated that transition interventions based on education and symptom management can improve quality of life and reduce symptom burden in patients with lung cancer. Although our design did not strictly constitute a transition intervention, the educational component and continuity of care from hospital to home reflect similar principles, thereby reinforcing the relevance of this type of approach.

Another noteworthy finding was the absence of hospital readmissions in the experimental group during follow-up, which may be attributed to the greater autonomy and functional capacity achieved. This result aligns with the evidence provided by Liang et al. [24], who found a reduction in postoperative complications and readmission rates following a structured pulmonary rehabilitation programme.

Moreover, the correlation analyses between functionality, quality of life, and general condition showed statistically significant positive associations, reinforcing the hypothesis that functional improvement is closely related to a more favourable perception of overall wellbeing, as previously suggested in the longitudinal study by Chen et al. [25]. This relationship is particularly relevant in the context of palliative and rehabilitative care, where the primary goal is to maximise quality of life rather than achieve disease cure—an assertion also supported by studies such as that by Lakkadsa et al. [26].

Compared with other intervention models, such as the post-COVID-19 telerehabilitation approach studied by Calvo-Paniagua et al. [27], our face-to-face and home-based protocol achieved similar impacts in variables such as dyspnoea, autonomy, and perceived exertion. This opens the door for future exploration of hybrid combinations of both approaches.

These results can also be compared with similar studies by Manocchio et al., in which personalised rehabilitative interventions were implemented from the early stages of hospitalisation, aiming to prevent functional decline and enhance recovery. The findings showed significant improvements in respiratory capacity, mobility, and patient autonomy among those who received early rehabilitation, compared to those who did not. This highlights the importance of implementing early rehabilitation strategies in acute hospital settings [28].

One of the strengths of the present study is its robust methodological design, featuring randomisation, multi-level blinding, and the use of validated tools to assess functionality and quality of life. In addition, the intervention was structured according to standardised criteria, which supports its replicability.

However, this study is not without limitations. The sample was small and drawn from a single hospital centre, which may restrict the generalisability of the findings. Furthermore, the follow-up period was limited to one month, and as such, the medium- and long-term sustainability of the observed effects remains uncertain. Future multicentre studies with longer follow-up periods and cost-effectiveness analyses may provide further insights into the feasibility of incorporating the ERP into routine clinical practice. We also consider that a one-month follow-up period is somewhat limited for drawing significant clinical conclusions. However, the clear short-term mortality risk in some participants led us to opt for this type of medium-term follow-up.

### Clinical Implications

The findings of this study have direct clinical implications. The ERP can be integrated as part of the interdisciplinary approach within oncology inpatient units, offering a structured, safe, and effective tool to enhance functionality in patients with associated respiratory conditions. The combination of education, energy conservation techniques, and direct functional re-education enables individualised care and empowers patients in their recovery process. Its implementation does not require complex equipment or substantial financial investment, which facilitates adaptation at different levels of care, including district hospitals and home-based services. This intervention model may also be incorporated into active palliative care protocols, where improving quality of life is a fundamental goal.

## 5. Conclusions

This clinical trial demonstrates that the Effort Re-education Programme is an effective intervention for improving functionality, autonomy, and quality of life in oncology patients with respiratory involvement. The results suggest that incorporating such programmes into routine clinical practice could represent a paradigm shift in oncology inpatient care, moving from a model focused solely on pharmacological treatment to a more comprehensive and functional approach.

In a healthcare context increasingly characterised by chronicity, frailty, and loss of autonomy, the promotion of early rehabilitative strategies such as the ERP not only improves clinical outcomes but also dignifies the care process. Supporting ill individuals in regaining—even partially—their everyday independence is also a means of relieving suffering and humanising care.

## Figures and Tables

**Table 1:** Demographic and clinical data baseline assessment.

Variables		IG (N = 33)	CG (N = 32)
Age		66.24 (±12.15)	64.59 (±14.9)
Sex	Male	63.6%	59.4%
	Female	36.4%	40.6%
Pathological diagnosis	Breast	21.2%	9.4%
	Lung	45.5%	43.8%
	Digestive system	6.1%	9.4%
	Pancreas	6.1%	6.3%
	Prostate	3%	9.4%
	Other	18.2%	18.8%
Number of oncology treatment lines		3 (1)	3 (4)
Barthel		58.12 (±19.78)	55.03 (±21.34)
EORTC QLQ-C30		58.40 (±14.25)	59.90 (±13.95)
Karnofsky		60.00 (±10.23)	62.50 (±11.12)
Lawton–Brody		3.10 (±1.52)	3.32 (±1.67)
Hospitalised time		11 days	12 days
Metastasis	Yes	75.8%	81.2%
	No	24.2%	18.8%
Hospital readmission	Yes	3%	37.5%
	No	97%	62.5%

**Table 2:** Pre-intervention, post-intervention, and change scores for the variables under study.

Variables	Intervention Group (IG) (n = 33)		Control Group (CG) (n = 32)		*p*-Value	Cohen’s d
	Basal Assessment (T0)	Final Assessment (T2)	Basal Assessment (T0)	Final Assessment (T2)		
Barthel	58.12 (±19.78)	76.45 (±17.62)	55.03 (±21.34)	61.22 (±20.08)	<0.001	0.730
EORTC QLQ-C30	58.40 (±14.25)	74.80 (±13.71)	59.90 (±13.95)	66.50 (±14.02)	<0.001	0.720
Karnofsky	60.00 (±10.23)	78.75 (±9.87)	62.50 (±11.12)	68.10 (±10.55)	<0.001	1.190
Lawton Brody	3.10 (±1.48)	5.88 (±1.60)	3.32 (±1.52)	4.10 (±1.67)	<0.001	0.880

## Data Availability

Upon study completion, the data will be made available via FAIRsharing on re3data.org (accessed on 10 March 2025).

## Supplementary Materials

The following supporting information can be downloaded at https://www.mdpi.com/article/10.3390/jcm14134417/s1: Figure S1: CONSORT flow diagram of participants included in the trial; S2: Clinical trial registration document; S3: Favourable opinion of bioethical committee; S4: Patient Information Sheet (HIP) and Informed Consent (IC); S5: Proof of financing.

## Abbreviations

The following abbreviations are used in this manuscript:

NCCN	National Comprehensive Cancer Network.
CAUSA	University Hospital Complex of Salamanca.
UDATO	Teaching and Clinical Unit of Occupational Therapy.
HRQoL	Health-related quality of life.
IADLs	Instrumental activities of daily living.
