# Supplementary material for: A Home-Based Interdisciplinary Intervention to Enhance Functionality in Oncology Patients: Results from a Clinical Trial
Source: J Clin Med. 2025 Jun 20;14(13):4417. doi: 10.3390/jcm14134417 (PMC12250040; doi:10.3390/jcm14134417)
Supplement: Supplementary file 1 [file jcm-14-04417-s001.zip › jcm-3694360-supplementary.pdf]

Figure S1. CONSORT flow diagram of participants included on the trial

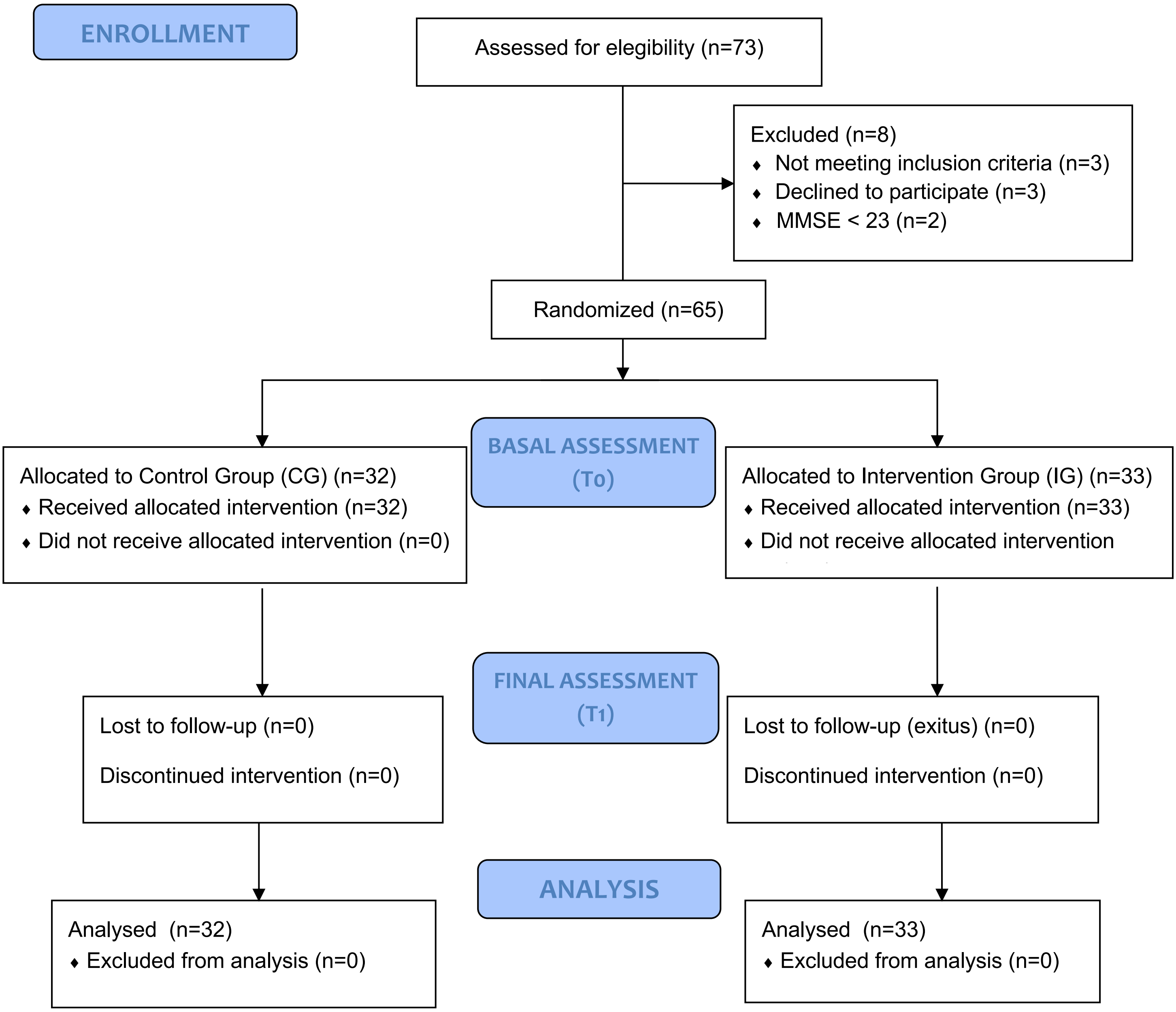

ClinicalTrials.gov PRS
Protocol Registration and Results System

ClinicalTrials.gov Protocol Registration and Results System (PRS) Receipt
Release Date: November 1, 2023

ClinicalTrials.gov ID: NCT06035263

Study Identification

Unique Protocol ID: University of Salamanca 2

Brief Title: Effects of a Functional Re-education and Environmental Adaptation Programme in Cancer Patients With Associated Respiratory Pathology

Official Title: Effects of a Functional Re-education and Environmental Adaptation Programme on Levels of Dependency, Dyspnoea, Functional Capacity, Quality of Life and Caregiver Burden in Cancer Patients With Associated Respiratory Pathology

Secondary IDs:

Study Status

Record Verification: November 2023

Overall Status: Not yet recruiting

Study Start: January 1, 2024 [Anticipated]

Primary Completion: December 31, 2024 [Anticipated]

Study Completion: December 31, 2024 [Anticipated]

Sponsor/Collaborators

Sponsor: University of Salamanca

Responsible Party: Principal Investigator
Investigator: Prof. Dr. Eduardo J Fernández Rodríguez [efernandezrodriguez]
Official Title: Professor
Affiliation: University of Salamanca

Collaborators:

Oversight

U.S. FDA-regulated Drug: No

U.S. FDA-regulated Device: No

U.S. FDA IND/IDE: No

Human Subjects Review: Board Status: Approved
Approval Number: 2022/10
Board Name: COMITÉ de Ética de la Investigación
Board Affiliation: University of Salamanca
Phone: 923291100
Email: comite.etico.husa@saludcastillayleon.es
Address:

Paseo de San Vicente, 58-182
37007 Salamanca
Comité Ético de Investigación con Medicamentos

Data Monitoring: No

FDA Regulated Intervention: No

Study Description

Brief Summary: the investigators propose an interdisciplinary intervention, carried out by occupational therapists, nurses, physiotherapists and doctors specialised in this type of patient, aimed at improving conventional clinical practice and implementing a programme of functional re-education and environmental adaptation that implements conventional clinical practice, and which the investigators also consider to be an essential intervention in the follow-up of patients with associated respiratory pathology once they are discharged from hospital.

Detailed Description: In recent years, improvements in oncological treatments, together with a strong commitment to preventive strategies, have led to better early diagnosis and better knowledge of the oncological pathology itself, resulting in an exponential increase in the survival of cancer patients. All of this has led to the increasing importance of the concept of "long survivorship".

Along with this increase in survival and the resulting increase in the number of lines of treatment used, there has been an increase in side effects that negatively impact aspects such as functionality or quality of life in patients. Some of these effects may include tumour asthenia, anxiety or associated respiratory pathology (dyspnoea). In some patients with advanced cancer, dyspnoea may be a clinical sign of end-stage disease. Approximately 41% of palliative care patients have dyspnoea and 46% of these describe it as moderate or severe. Furthermore, this incidence increases significantly in patients with an anatomopathological diagnosis of lung cancer, reaching incidences of more than 73% in various studies.

Most patients perceive this dyspnoea as a limiting factor beyond their control, leading to avoidance behaviours that further increase their inactivity. This inevitably has a negative impact on their functionality and leads to patterns of fear/avoidance of exercise in cancer patients, as seen in patients with chronic pain, chronic fatigue syndrome or fibromyalgia. Patients with respiratory problems adapt to their symptoms by reducing their activity levels. This leads to a deterioration in physical fitness and exertional dyspnoea, known as the "respiratory patient cycle".

For the control of dyspnoea, the investigators believe that the measures used in conventional clinical practice, developed mainly from a pharmacological point of view, can be implemented, but that they are not sufficient to achieve optimal symptomatic control.

Current pharmacological treatment does not always manage to control the main symptom of respiratory pathologies: dyspnoea, perhaps because of its multifactorial nature. Most patients perceive this symptom as a limiting factor beyond their control, leading them to adopt avoidance behaviours that increase their inactivity, with negative consequences for their functionality. Therefore, from the point of view of comprehensive care for the patient, not only for the symptom, the investigators have observed that it is necessary to use other interventions that achieve the patient's readaptation to their daily activity.

the investigators have observed that cancer patients with respiratory pathology adapt to their symptoms by reducing their level of activity. This leads to a deterioration in their physical condition and dyspnoea on exertion. Dyspnoea leads to progressive disability with loss of mobility, self-esteem, work and social relationships.

These data show that associated respiratory pathology is a common problem in oncology, which is still underestimated by professionals. It has been observed that people with this process find it difficult to normalise their daily lives, either because of a deterioration in their clinical condition or because of a problem in generalising what they have learned during their hospital stay.

In terms of intervention, the NCCN panel considers education and energy conservation techniques to be fundamental, always within a complete functional rehabilitation programme.

In addition to educational measures, the NCCN panel recommends the prescription of energy conservation techniques, presenting them as useful in controlling this associated respiratory pathology. To this end, the panel analyses, on the one hand, a multicentre clinical trial involving 296 patients currently under active treatment, in whom a reduction in symptom intensity was reported after application of the programme. On the other hand, they present a meta-analysis including 113 studies with a total sample of 11,525 patients, showing an improvement in individuals after the use of non-pharmacological measures to control associated respiratory pathology (weighted effect size, 0.30; 95% CI, 0.25-0.36; $p<0.001$).

The common presence of associated respiratory pathology in cancer patients is a factor that can cause changes in body composition, such as loss of muscle mass or an increase in visceral fat. This type of non-pharmacological intervention has been shown to be an important tool in improving symptoms and some parameters related to body composition. In the study by Madison et al, moderate exercise (bioelectrical impedance) over 12 weeks was associated with a reduction in body fat in colorectal cancer survivors. Another study showed that an 8-week exercise intervention increased skeletal muscle mass and decreased visceral fat in a group of head and neck cancer patients undergoing chemotherapy. Fernandez-Lao et al showed that a multimodal exercise programme in breast cancer patients led to a reduction in body fat and an increase in lean body mass in a group of breast cancer patients. However, despite the potential effects, there is little evidence of functional rehabilitation interventions that have assessed the effects on body composition in patients with associated respiratory pathology.

The scientific evidence and recent conclusions of expert meetings on cancer and the benefits of these psychosocial interventions, both in the prevention and treatment of the different clinical aspects of cancer survivors or patients undergoing cancer treatment, point to the existence of sufficient evidence to support their efficacy in addressing physical function, fatigue, quality of life, pain, anxiety and depressive symptoms, among others, related to cancer.

Non-pharmacological psychosocial interventions have therefore been shown to be even more effective than pharmacological interventions in addressing these associated symptoms, leading us to consider the bio-psychosocial approach and multidisciplinary intervention (oncology, nursing, physiotherapy, occupational therapy and medicine) as the global context of intervention. For this reason, aspects related to loss of function and pain associated with anxiety-avoidance disorders should also be assessed and addressed, which can be assessed using kinesiophobia scores.

The cognitive-behavioural model of fear of exercise suggests that patients with chronic pain or fatigue syndrome tend to avoid activity because they believe that activity is the cause of these symptoms, such as pain and fatigue.

Avoidance behaviour leads to even greater fear and symptoms, resulting in more pain or fatigue, which may extend to patients with associated respiratory pathology, so it is important to restore optimal activity levels and avoid loss of physical function and ability.

The choice of the most appropriate intervention setting is based on clinical complexity and the patient's ability to self-manage their situation. For this reason, and with the support of experts, our study proposes a supervised intervention in the home setting after hospital discharge, adapted to the specific situation of patients with associated respiratory pathology who have just been discharged from hospital. This is in line with recommendations to improve and increase access and adherence to a functional rehabilitation programme for these patients.

Supervised follow-up outside the healthcare setting, both in the community and at home, has been shown to be successful in previous trials. This is shown in a meta-analysis of 14 randomised controlled clinical trials in breast cancer survivors with supervised intervention by telephone or e-mail. In any case, despite the choice of the home setting for its feasibility in our study population, supervision and controlled follow-up of the intervention is still a guarantee of good results, as shown in another recent meta-analysis of 128 trials with a total of 13,050 cancer patients, where supervised programmes had greater effects on physical activity. Follow-up is important not only for the correct implementation of the intervention programme, but also for achieving high adherence to the programme, as shown in a review of 23 trials and 1372 patients.

The best results in patients surviving or undergoing cancer treatment have been obtained with multimodal exercise programmes that combine different types of exercise, mainly aerobic and strength training, in addition to other interventions such as reeducation in activities of daily living and health education, adapted to the patient's general condition and functional capacity.

For this reason, the investigators propose an interdisciplinary intervention, carried out by occupational therapists, nurses, physiotherapists and doctors specialised in this type of patient, aimed at improving conventional clinical practice and implementing a programme of functional re-education and environmental adaptation that implements conventional clinical practice, and which the investigators also consider to be an essential intervention in the follow-up of patients with associated respiratory pathology once they are discharged from hospital.

Conditions

Conditions: Cancer
Dyspnea
Functionality
Quality of Life
Functional Capacity

Keywords: Functional Status
Cancer
Dyspnea
Quality of Life
Occupational therapy

Study Design

Study Type: Interventional

Primary Purpose:	Supportive Care
Study Phase:	N/A
Interventional Study Model:	Parallel Assignment
Number of Arms:	2
Masking:	Single (Participant)<br>Subjects will also be blinded and will not know which group they have been allocated to and therefore which intervention they will receive. In order to minimise any contamination between groups, the assessment process will be carried out by external research staff who will perform the measurements and who have been previously trained and educated to avoid subjective bias in the process, as they will be unaware of the intervention group to which each elderly centre is assigned, so the clinical trial will have a masking of the blinded assessment by third parties.
Allocation:	Randomized
Enrollment:	80 [Anticipated]

Arms and Interventions

Arms	Assigned Interventions
Experimental: FUNCTIONAL REEDUCATION AND ENVIRONMENTAL ADAPTATION PROGRAMME<br>prescription of reeducation in activities of daily living and the prescription of assistive devices and environmental adaptations, for one month from the first baseline assessment at the time of hospital discharge.	FUNCTIONAL REEDUCATION AND ENVIRONMENTAL ADAPTATION PROGRAMME<br>the prescription of reeducation in activities of daily living and the prescription of assistive devices and environmental adaptations, for one month from the first baseline assessment at the time of hospital discharge.
Active Comparator: Health education programme:<br>instructions and recommendations for maintaining an active and healthy life will be given as part of a health education programme. These will focus on the benefits of an active lifestyle and general guidelines to follow, as well as the importance of nutrition and hydration in a healthy lifestyle.<br>They will receive the dossier of instructions and recommendations of the health education programme, as in the experimental group.	Health education programme:<br>instructions and recommendations for maintaining an active and healthy life will be given as part of a health education programme. These will focus on the benefits of an active lifestyle and general guidelines to follow, as well as the importance of nutrition and hydration in a healthy lifestyle. They will receive the dossier of instructions and recommendations of the health education programme, as in the experimental group.

Outcome Measures

Primary Outcome Measure:

1. Activities of daily living
   Barthel Index

   [Time Frame: Baseline; 1 week (follow up); 2 weeks (final)]

Secondary Outcome Measure:

2. dyspnoea
   Medical Research Council Dyspnoea: Scores: (0, best - 4, worst)

   [Time Frame: Baseline; 1 week (follow up); 2 weeks (final)]

3. Health-related quality of life
   EuroQol 5-D questionnaire. Scores: ranging from 0 (worst imaginable state of health) to 100 (best imaginable state of health).

[Time Frame: Baseline; 1 week (follow up); 2 weeks (final)]

4. General Pain
   Visual Analogue Scale. Scores: ranging from 0 (lowest degree of pain) to 10 (highest degree of pain).

   [Time Frame: Baseline; 1 week (follow up); 2 weeks (final)]

5. Physical performance assessment
   Short Physical Performance Battery. The total SPPB score is the sum of the three sub-tests and ranges from 0 (worst) to 12. Changes of 1 point have clinical significance. A score below 10 indicates frailty and an elevated risk of disability as well as falls.

   [Time Frame: Baseline; 1 week (follow up); 2 weeks (final)]

6. pain/fatigue-related fear of movement
   Tampa Scale for Kinesiophobia. The results are obtained by means of a total raw score (which can range from 17 to 68) and two subscale scores.

   [Time Frame: Baseline; 1 week (follow up); 2 weeks (final)]

Eligibility

Minimum Age: 18 Years

Maximum Age:

Sex: All

Gender Based: No

Accepts Healthy Volunteers: No

Criteria: Inclusion Criteria:

- To have, among the reasons for admission, an anatomopathological diagnosis of newly diagnosed or relapsed oncological disease.
- To be admitted to the Oncology Department of the University Hospital of Salamanca.
- Moderate to severe dependency: Barthel Index score between 20 and 55 points.
- Sign an informed consent form authorising their voluntary participation in the study.

Exclusion Criteria:

- Cognitive impairment as assessed by the Mini-Mental State Examination (MMSE) of less than 24 points.
- Haemoglobin level of less than 10g/dl.

Contacts/Locations

Central Contact Person:

Central Contact Backup:

Study Officials:

Locations: **Spain**
Eduardo Jose Fernandez Rodriguez
Salamanca, Castilla Y Leon, Spain, 37002
Contact: Eduardo PhD Fernandez, PhD 923294500 edujfr@usal.es

IPDSharing

Plan to Share IPD:

References

Citations:

Links:

Available IPD/Information:

Subscribe to DeepL Pro to translate larger documents.
Visit www.DeepL.com/pro for more information.

COMPLEJO ASISTENCIAL UNIVERSITARIO D SALAMANCA
Paseo de San Vicente, 58-182
37007 Salamanca
Medicines Research Ethics Committee
Telephone: 923 29 11 00 - Ext. 55 515

CEIm
ÁREA DE SALUD DE SALAMANCA

Sacyl

E-mail: comite.etico.husa@saludcastillayleon.es

OPINION OF THE COMMITTEE ON THE ETHICS OF MEDICINAL PRODUCTS RESEARCH

Ms. CONCEPCIÓN TURRIÓN GÓMEZ, Technical Secretary of the Salamanca Health Area Research Ethics Committee,

C E R T I F I C A

That this Committee, at its meeting on 24/10/2022CEIm Ref. 2022/10
has assessed the Research Project entitled

EFFECTS OF A SPECIFIC FUNCTIONAL REHABILITATION PROGRAMME IN ONCOLOGY PATIENTS WITH RESPIRATORY PATHOLOGY: DYSPNOEA

CEIm Code: PI **2022 09 1150**

of which he is Principal Investigator Ms María Isabel Rihuete Galve

of the Medical Oncology Medical Oncology

assessed in accordance with Law 14/2007 on Biomedical Research, the ethical principles of the Declaration of Helsinki of the World Medical Association on ethical principles for medical research involving human subjects, as well as the other ethical principles and legal regulations applicable according to the characteristics of the study,

Considers that this study meets the necessary requirements and is feasible to be carried out in this centre, and therefore, it is hereby **APPROVED** for this study to be carried out.

And for the record, this is signed at Salamanca, dated 07 November 2022 THE

SECRETARY

TURRION GOMEZ MARIA DE LA CONCEPCION - 07986126C

Digitally signed by TURRION GOMEZ MARIA DE LA CONCEPCION - 07986126C
Date: 2022.11.07 13:53:59 +01'00'

S.D.: Ms. Concepción Turrión Gómez

Composition of the CEIm of the Salamanca Health Area
Chairman: D. Luis Muñoz Bellvís (Head of the General Surgery and Digestive System Department) Vice-president: Mr. Enrique Nieto Manibardo (Data Protection Delegate of CAUSA)
Secretary: Ms. Concepción Turrión Gómez (Pharmacist and Biochemist - Representative of the Scientific Committee - IBSAL). Members: Mr. Ricardo Tostado Menéndez (Clinical Pharmacologist); Ms. Silvia Jiménez Cabrera (Hospital Pharmacy); Ms. Ascensión Hernández Encinas (Professor of Applied Mathematics, University of Salamanca. President ASCOL, patient representative); Ms. Mª Teresa Arias Martín (Mental Health Nurse. Member of the Healthcare Bioethics Committee); Ms. Mª del Carmen Arias de la Fuente (Clinical Trials Management Technician); Ms. Berta Bote Bonaechea (Psychiatry Specialist); Ms. Ángela Rodríguez Rodríguez (Head of Nursing Unit. Haematology Department); Mr. Guzmán Franch Arcas (Head of the Haematology Department); Ms. Guzmán Franch Arcas (Clinical Trials Management Technician); Ms. José María de la Fuente (Clinical Trials Management Technician). Guzmán Franch Arcas (Specialist in General Surgery and Digestive System); Mr. Antonio Márquez Vera (Physiotherapist); Ms. Ana Martín García (Specialist in Cardiology); Ms.

Teresa Martín Gómez (Specialist in Oncology); Ms. Concepción Rodríguez Barrueco (Primary Care Pharmacist); Mr. Manuel Ángel Gómez Marcos (Primary Care Doctor. Head of the Primary Care Research Unit in Salamanca), Ms. Belén Vidriales Vicente (Head of Haematology Section).

Subscribe to DeepL Pro to translate larger documents.
Visit www.DeepL.com/pro for more information.

PATIENT INFORMATION SHEET (HIP) AND INFORMED CONSENT (CI)

Title of the study

EFFECTS OF A SPECIFIC FUNCTIONAL REHABILITATION PROGRAMME IN ONCOLOGY PATIENTS WITH RESPIRATORY PATHOLOGY: DYSPNOEA

Introduction

We are writing to inform you about a research study in which you are invited to participate. Your participation is voluntary. Please take the time to read the following information and ask any questions you may have. Ask the researcher of this study if there is anything that is not clear to you or if you would like more information.

Aim of the study

The main objective is to test the efficacy of the implementation of a Functional Rehabilitation Programme on the quality of life of oncology patients with respiratory pathology.

This research study has been approved by the Salamanca Research Ethics Committee (Comité de Ética de la Investigación con Medicamentos de Salamanca).

Study procedures and potential risks and discomforts

Once you have given your consent and the researcher has verified that you meet the criteria for participation in this study, a visit lasting approximately half an hour will consist of questions about your health and ability to carry out certain aspects of your daily life and an assessment to determine the presence of signs of frailty. In addition, the state of your cognitive performance will be examined. These measurements will also be taken at the time of discharge from hospital.

After the first visit, participants will be randomly distributed into two groups: Group 1: Participants in this group will continue to receive their usual care within the clinical process.

Group 2: Participants assigned to the experimental group, in addition to the usual clinical practice, will undergo a Functional Rehabilitation Programme during their hospital stay. This programme includes Prescription of multimodal physical exercise to be performed daily in two short sessions of 15-20 minutes, one in the morning and one in the afternoon, re-education in activities of daily living and Prescription of support products and environmental adaptations, according to each context.

Participation in this study would not cause any discomfort, and does not involve any health risk.

Other data about you relevant to this research study will be stored in coded form. All information generated in this study will be used exclusively for the purposes specified here.

All the information on this study will be stored in coded form, and will be used exclusively for the purposes specified herein. In the event that your data are transferred to other research groups, this will always be done in accordance with current legislation, keeping your data coded, in order to carry out studies related to the objectives of this work, and with prior authorisation from the Salamanca Research Ethics Committee on Research with Medicinal Products. In the event that the objectives of the research work proposed by other research groups are different from those of the present project, a new consent will be requested.

Voluntary participation and withdrawal
You are free to decide whether or not you wish to take part in this study, participation is completely voluntary. If you decide to participate, you still have the possibility to withdraw at any time, without having to give any explanation, and without any penalty or negative consequences for you. If you change your mind about your samples or your data, you have the right to request their destruction or anonymisation, through your doctor. However, you should be aware that the data obtained from the analyses carried out up to that point may be used for the purposes requested and may be retained in compliance with the corresponding legal obligations.

Potential benefits
No direct benefit is expected from your participation in the study. However, the information obtained from this research project may contribute to medical progress and could help other patients in the future. You will not receive any financial benefit from the release of the data provided, nor will you have any rights to potential commercial benefits from any discoveries that may be made as a result of the research conducted.
The benefit for you will be to learn in greater depth about aspects related to the improvement of your life in the context of the clinical process. You will be informed of the results of the examinations and evaluations carried out. The examinations carried out do not entail any risk to life, only the discomfort that the duration of the tests (one and a half hours) may entail, none of which are invasive.

Data protection and confidentiality
All information about your results will be treated in the strictest confidence. Both the Centre and the research team are responsible for the processing of your data and undertake to comply with the data protection regulations in force, currently Organic Law 3/2018, of 5 December, on the Protection of Personal Data and guarantee of digital rights and Regulation (EU) 2016/679 of the European Parliament and of the Council of 27 April 2016 on Data Protection (RGPD). The data collected for the study will be identified by a code, so that no information that can identify you is included, and only the research team will be able to relate this data to you.
Your data will be analysed by the research team based on the public and/or legitimate interest in achieving the purposes of the study. Subsequently, your personal information will only be kept by the centre for your health care and by the research team for other scientific research purposes if

you have given your consent to do so and if permitted by applicable law and ethical requirements.
If the results of the study are likely to be published in scientific journals, no personal data of the participants in this research will be provided at any time. We inform you that you have the right to access, rectify or cancel your data, and you can limit the processing of data that are incorrect, request a copy or that the data you have provided for the study be transferred to a third party. To exercise your rights, or in the event that the participant wishes further information on the processing of your personal data, you may contact the principal investigator of the study whose details are specified at the end of this document, the Data Protection Officer of the Regional Health Authority (dpd@saludcastillayleon.es) or our centre (dpd.husa@saludcastillayleon.es). You also have the right to contact the Data Protection Agency if you are not satisfied.

Information on results
At your request, at the end of the study and in accordance with article 27 of Law 14/2007 on Biomedical Research, you may be provided with information on the results of this research work.

Contact details of the research team:
If you have any questions about the study or any aspect of it, please contact the principal investigator, Dr. Eduardo José Fernández Rodríguez, at 923291100 ext.55112.

Version 1
26 October 2022

INFORMED CONSENT FORM (CI)

I *(Name and Surname)* ____
I was able to ask questions about the study.
I have received sufficient information about the study.
I have read the information sheet provided to me.
I have spoken to the Investigator __________________________
I understand that my participation is voluntary. I
understand that I may withdraw from the study:

1st Whenever you want

2nd Without having to give explanations

3º Without having any negative repercussions

I voluntarily agree to participate in the Project and authorise the use of all information obtained. I understand that I will receive a signed copy of this informed consent.
I authorise the project managers to use my data for the purposes and in the manner described in this document.

☐ Yes ☐ No

Participant's signature Date

Name and signature of the researcher Date

Version 1

26 October 2022

Subscribe to DeepL Pro to translate larger documents.
Visit www.DeepL.com/pro for more information.

COLLABORATION AGREEMENT

In Madrid,

MEETING

On the one hand, **Bristol-Myers Squibb S.A.U.**, domiciled at Calle Quintanadueñas 6, 28050 Madrid, Spain, with tax identification number B-84416130, and in its name and on its behalf **Mr. Roberto Urbez Plasencia**, in his capacity as attorney-in-fact of the same, by virtue of General Manager (hereinafter, "**BMS**").

And on the other part, the **Fundación Instituto de Estudios de Ciencias de la Salud de Castilla y León (IECSCYL), in its capacity as managing body of the Instituto de Investigación Biomédica de Salamanca (IBSAL)**, with registered office for notification purposes at the Complejo Asistencial Universitario de Salamanca, Paseo de San Vicente, 58-182, 37007 Salamanca, and in its name and on its behalf**, María de Lorenzo Santiago**, DNI **07.969.495-H**, in her capacity as **Director of Management of the IBSAL** (hereinafter, "the **Foundation**").

With the VºBº and Conformity of Dr. Emilio Fonseca Sánchez with NIF 07824069K and domiciled for these purposes at the Complejo Asistencial Universitario de Salamanca, Servicio de Oncología (hereinafter the "**Project Manager**").

Both parties recognising that they have sufficient legal capacity, have signed this document and, to this effect,

EXHIBIT

I. Bristol-Myers Squibb is a biopharmaceutical company dedicated to the research, development and distribution of biopharmaceutical products in various therapeutic areas and is particularly interested in the area of oncohaematology and immunotherapy through the development of collaborative projects with hospitals and healthcare centres that help to improve the quality of life of patients, among other issues.

II. That the **Institute of Biomedical Research of Salamanca (IBSAL)**, managed through the Foundation Institute of Health Sciences Studies of Castilla y León (IECSCYL), is an entity dedicated to promoting and carrying out medical research in the **University Health Care Complex of Salamanca** as a basic nucleus, and that its management entity is included in the list of non-profit entities provided for in article 16 of Law 49/2002 on the tax regime for non-profit entities and tax incentives for patronage. In this sense, the IBSAL constitutes the research management structure of the Complejo Asistencial Universitario de Salamanca and therefore, through the Foundation, has the capacity to sign this Agreement on behalf of the **Complejo**

Asistencial Universitario de Salamanca, where the Project that is the object of this Agreement will be developed.

III. Those obligations that the Foundation acquires in the present Agreement shall also be understood as obligations of the Hospital insofar as they affect it and with respect to the researchers belonging to the Hospital.

IV. That the Project Manager is a healthcare professional specialising in the field of oncohematology and has all the necessary authorisations to be able to carry out the project in the **University Healthcare Complex of Salamanca.**

V. That the Foundation has the necessary technical and human resources and wishes to collaborate with BMS under the terms set out in this Agreement and in accordance with the following

CLAUSES

<u>FIRST </u>- OBJECTIVE

The parties agree to collaborate jointly in the development of the "PASOS" project (hereinafter, the "**Project**"), a project of Functional Rehabilitation of oncology patients with associated respiratory pathology, supervised by Dr. Fonseca, doctor of the Oncology Service of the **Complejo Asistencial Universitario de Salamanca** and Head of the Project (hereinafter, the "**Project**").

The aim of the project is to improve the respiratory capacity of cancer patients with associated respiratory pathology. To this end, the IBSAL, through the Project Manager, will design together with BMS the functional rehabilitation programme to implement it in the Complejo Asistencial Universitario de Salamanca and subsequently, depending on the benefit provided to these patients, the extension of the Project, via training workshops, to other hospitals where the need is detected, will be assessed.

A detailed description of the Project is set out in **Annex I** attached to this Agreement. In particular:

(i) object, purpose and content of the Project;

(ii) actions that the parties undertake to carry out in the framework of the Project;

(iii) timetable for the implementation of the project;

(iv) measuring the results of the project.

This Collaboration Agreement complies with the provisions of article 34 of Law 14/2011, of 1 June, on Science, Technology and Innovation, and articles 47 and following of Law 40/2015, of 1 October, on the Legal Regime of the Public Sector.

<u>SECOND </u>- ECONOMIC CONDITIONS

BMS is committed to collaborate and contribute to the financing of the Project that will be carried out by

under this Agreement, for a total amount of fifteen thousand euros (€15,000), not including applicable taxes, where applicable.

The breakdown of the items to be covered by the above-mentioned financial contribution, as well as the terms of payment, are detailed in **Annex II**.

The parties acknowledge that the payment of the amounts provided for in this clause are considered adequate for the development of the activities contemplated within the framework of the Project, without at any time exceeding the total costs associated with the same, and do not constitute an incentive for the recommendation, prescription, purchase, supply, sale or administration of products owned by BMS and also declare that the delivery of the financial contribution does not entail any obligation to prescribe, supply, recommend or purchase BMS products under a preferential status.

Likewise, the Foundation guarantees that the economic contribution received by virtue of this Agreement will be destined to the realisation of the Project that is the object of the collaboration. Under no circumstances may the amount received be used to finance, directly or indirectly, in whole or in part, any activity that does not have a content directly related to the purpose of the collaboration (i.e. recreational activities, tourist visits, sporting activities). In the event of non-compliance with the provisions of this paragraph, BMS reserves the right to terminate this Agreement and the Foundation shall reimburse BMS in full for any amounts received under this Agreement.

THIRD.- M O N I T O R I N G COMMITTEE

The Parties shall set up a Monitoring Committee which shall meet, in person or virtually, to monitor this Agreement and ensure proper coordination of the actions of the Parties, which shall be composed of the following members:

- A person designated by the Foundation: María de Lorenzo Santiago, or person delegated by her.
- The Project Manager: Emilio Fonseca Sánchez
- A BMS appointee: Patricia de la Concha

The Monitoring Committee shall meet at least twice a year and shall meet for the first time three months after the signature of this Agreement. The Commission may be convened by any member, with a minimum of fifteen days' notice, if necessary to discuss any matter for the benefit of the execution of this Agreement.

In general, the functions of this Commission shall be

- Discuss and comment on the implementation of the project;
- Monitoring of the use of the funds provided;
- Monitoring of the indicators and results of the project;
- Analyse Ensure and propose the measures necessary for the best fulfilment of the purposes of this Agreement;
- Resolve any conflict, dispute or incident that occurs during the implementation of the Project; and

- Produce a final report summarising the main conclusions of the project from the meetings.

FOURTH - OBLIGATIONS OF THE PARTIES

The parties undertake to carry out the Project in accordance with the terms and conditions indicated in this Agreement and its annexes, and in strict compliance with the applicable regulations and sectoral codes, including Farmaindustria's Code of Good Practice for the Pharmaceutical Industry and the transparency obligations referred to in Clause Eleven.

Furthermore, the parties undertake to:

i. immediately notify the other Party of any information relating to the Project that could substantially affect the normal development of the Project.

ii. take all necessary and reasonable measures to perform its assigned tasks in the work programme of the Project in time, and to make information and results available to the other party in time, where this is necessary for the normal progress of the Project.

iii. ensure the accuracy of any information or materials you provide to the other party and correct as soon as possible any errors in such information or materials of which you have been notified. The receiving party is responsible for its use of such information and materials.

iv. act at all times in good faith and in a manner that reflects the good name, goodwill and reputation of the Parties, in accordance with sound ethical business, medical and health care practices.

v. participate collaboratively in the meetings set up by the Project's coordinating bodies.

For its part, the Foundation and the Project Manager undertake to:

i. collaborate with BMS in the definition, development and execution of the Project.

ii. collaborate with BMS in the analysis of the Project indicators.

iii. comply with the applicable regulations as well as with the internal policies of BMS applicable to the Project and communicated for this purpose.

iv. obtain and maintain all authorisations, licences and permits necessary for the realisation of the Project.

v. to have at all times during the term of the Agreement the facilities, experience and specific technical knowledge necessary for the correct development of the Project.

vi. to allocate the amounts agreed in Clause Two exclusively to the realisation of the Project.

vii. be responsible for all aspects related to the development of the Project that correspond to it, including the hiring and direction of the personnel necessary for the realisation of the Project.

viii. attend the Monitoring Committee defined in Clause Three. For its

part, BMS undertakes to:

ix. collaborate with all parties in the definition, development and implementation of the Project.

x. obtain and maintain all authorisations, licences and permits necessary for the realisation of the Project.

xi. to have at all times during the term of the Agreement the experience and specific technical knowledge necessary for the correct development of the Project.

xii. comply with the applicable regulations as well as with the Company's internal policies applicable to the Project and communicated to it for this purpose.

xiii. attend the Monitoring Committee as defined in Clause Three.

xiv. comply with the pharmacovigilance obligations provided for in this Convention

FIFTH - NON-EXCLUSIVITY

The parties acknowledge and agree that this Agreement is entered into on a non-exclusive basis and nothing in this Agreement limits the parties' ability to enter into similar agreements with third parties.

SIXTH - TERM AND TERMINATION

This Agreement shall enter into force upon signature by the last of the parties and shall expire **12 months after its entry into force,** unless terminated earlier. Once the initial term has expired, the Agreement shall be automatically terminated without prejudice to its extension by express written agreement of both parties before its expiry.

The parties may terminate this Agreement at any time by written notice in the event of a serious breach by the other party, which is not remedied within fourteen days.
(14) days after receipt of written notice from the party in default identifying the default and its intention to terminate the Agreement if it is not cured. Either party may also unilaterally terminate the Agreement by

thirty (30) days' notice in writing.

The termination or early termination of the Agreement shall not affect rights already accrued and obligations already incurred by the parties (including payments due or committed at the date on which the termination or expiry takes effect) or the entry into force or continuation in force of the provisions of the Agreement which are expressly or impliedly to take effect after its expiry. To this effect, the parties undertake to fulfil the outstanding commitments and obligations until their full settlement and to return the materials, information and/or Equipment in their possession that are the property of the other party or to which it has any exploitation rights. In the event that the agreement is terminated for any reason and the Foundation has not used all the funds contributed by BMS to the Project, the Foundation shall return the unused remainder to the Company.

SEVENTH - CONFIDENTIALITY

The parties undertake to maintain the confidentiality of information communicated to them or otherwise obtained by them in connection with this Agreement. The foregoing obligation shall not apply to such information:

(a) is in the public domain (unless it is in the public domain as a result of a breach of this Agreement);

(b) is in the possession of the party who received it without breach of any duty of confidentiality; or

(c) must be disclosed by law or administrative provision or obligations relating to listed companies, or to comply with transparency obligations under self-regulatory codes applicable to the pharmaceutical sector.

This confidentiality undertaking shall enter into force on the same date as this Agreement and shall remain in force for a period of **five (5) years** after its termination.

EIGHTH - DATA PROTECTION

The parties undertake to comply with the provisions of national and European legislation applicable to personal data as regards the collection, use, communication, storage and processing of such personal data obtained in the framework of the implementation of this Agreement.

The parties shall be responsible for the processing of the personal data they respectively collect in execution of this Agreement, unless otherwise agreed, and undertake to indemnify and hold each other harmless for any damage or prejudice caused as a consequence of each party's failure to comply with the provisions of the aforementioned regulations.

The processing of the personal data of the signatories to the Agreement, as well as the proxies and employees of the parties, which are necessary for the proper execution of the Agreement, will be carried out in accordance with the provisions of the General Data Protection Regulation, for the

for the purposes of administration, management, archiving and control of the Agreement, for disclosure to the parties' subcontractors in the framework of the execution of the Agreement and for the fulfilment of the legally required obligations of the parties, for the duration of the Agreement and, thereafter, for the periods required for the fulfilment of the legal obligations of both parties.

In relation to the processing of the personal data referred to in the previous paragraph, in accordance with the provisions of the General Data Protection Regulation:

- data subjects may exercise their rights of access, information, portability, rectification, limitation, erasure and objection. In the case of BMS, the interested party may exercise these rights and request additional information on the processing of their personal data by sending their request to the following e-mail address: EUDPO@BMS.COM. In the case of the Foundation, by sending their request to the e-mail address [protecciondedatos@ibsal.es].

- Data subjects may also lodge a complaint with their competent supervisory authority if they consider that the processing of information by BMS or the Foundation is in breach of applicable law.

- BMS may also share personal data with other BMS affiliates or with service providers contracted by BMS for the purposes described above. These companies may be based in the European Union or in third countries. BMS will ensure adequate protection and lawful transfer of data between its group companies in accordance with data protection laws and will ensure that your personal information is adequately protected in compliance with applicable law where such companies are located in countries in respect of which an adequate level of protection for personal data may not have been declared by the European Commission.

- The Foundation may transfer the personal data of the signatories in the cases provided for by law and, where appropriate, to the organisations that make up the specific collaboration agreement for the creation of the IBSAL, signed between the Regional Ministry of Health of Castilla y León and the University of Salamanca signed on 21 March 2011 and its subsequent amendments.

NINTH - MATERIALS

Within the framework of this Agreement, the parties agree as follows:

The Project Leader and the Company shall prepare the Materials foreseen in Annex I of this agreement.

All Materials or content created for the Project created by the Foundation, through the Project Manager, will be subject to the Company's internal policies on review and approval of materials and may be adjusted as deemed necessary by the Company.

For the purposes of this Clause, "Materials" shall mean all documents, software or any other written material, whether in hard copy, electronic, audio-visual or any other format, whether in draft or final form, and any creation or output created or generated under this Agreement.

The Materials shall in no case contain any mention or reference, directly or indirectly, to specific medicinal products intended for the treatment of diseases, marketed or under investigation.

Under no circumstances may the Materials contain the logo, commercial name or any direct or indirect mention of the participation or sponsorship of any entity that is not a party to this Agreement, unless the parties expressly agree to do so and, in such case, the express consent of such entity or entities has been obtained.

TENTH - INDUSTRIAL AND INTELLECTUAL PROPERTY, USE OF NAMES AND PUBLICATIONS

This Agreement does not affect the pre-existing intellectual and industrial property rights of the parties, which shall remain the property of the party that introduced them. This means, by way of example but not limitation, that the parties shall continue to own their intellectual and industrial property rights in their logos, trade names, trademarks.

All the results and Materials that may be generated by the parties as a consequence of carrying out the Project that is the object of this Agreement shall be the property of the party that created them. However, each party assigns to the other party on a non-exclusive basis and with the right to transfer to third parties, exclusively or otherwise, for the entire term of protection established in the applicable legislation and for exploitation throughout the world, all intellectual and industrial property rights over the Materials generated for the Project, which may be freely exploited by the other party for as long as the duration of the rights over the results and Materials remain in force, throughout the world and without limitations of any kind. The rights granted under this Agreement include, but are not limited to, the rights of reproduction, public communication, distribution and transformation. Each party shall be entitled to exploit the Materials in any medium and by any system, means and process. The Parties represent and warrant that the provisions of this Clause do not infringe or affect the rights or interests of any third party outside of this Agreement.

Furthermore, the Parties acknowledge their individual rights to publish the results of the Project. In this sense, the parties may undertake any action aimed at publishing, disseminating, or presenting the results, including but not limited to oral conferences, doctoral theses, *abstracts* to congresses, scientific or divulgative articles, or any other form of communication intended in relation to the project, as long as this does not affect the protection of the information to be disclosed or affect the conditions of confidentiality, or as explicitly agreed in each Annex.

For this purpose, the party wishing to disclose any information related to the Project shall send with

a copy of the medium to be published to the other parties not less than forty-five (45) days in advance.

The other party must express its agreement or disagreement in writing to such a request within a maximum period of thirty (30) days from the date of receipt of the request, providing the other party to the Project with its comments and/or suggestions. The party receiving the suggestions shall endeavour to do its best to incorporate such suggestions. Each Party may only object to a publication in the event that it justifies that the publication contains confidential information of the same, erroneous information on the development of the Project or that it does not include the wording indicated below.

In any documentation, posters, propaganda or publications to be produced by the parties for dissemination or public disclosure in relation to the Project exclusively in the cases foreseen in this Agreement, special mention must be made of the parties. In relation to BMS, the publications shall include the following disclaimer "With the collaboration of BMS in the PASOS Project". In relation to the Foundation, publications will include the following disclaimer: "With the collaboration of the Institute of Biomedical Research of Salamanca (IBSAL)". The use of the name of any of the Parties shall require their prior express written authorisation.

ELEVENTH - BEST PRACTICES AND CONFLICT OF INTEREST

Each of the parties must comply with all legislation, regulations, rules and sectoral codes that are applicable to them according to their legal nature and scope of action, including the Code of Good Practice of the Pharmaceutical Industry, as well as national and international anti-corruption and anti-bribery regulations, such as the *U.S. Foreign Corrupt Practices Act,* which prohibits bribery and the payment or offering of money or objects of value to public officials or members of political parties for the purpose of obtaining business benefits. In the event of an open investigation of corrupt practices by the Foundation or the Project Leader, the Foundation shall inform BMS as soon as possible and BMS may unilaterally terminate this Agreement.

Furthermore, the Foundation and the Project Leader warrant that there is no conflict of interest that may prevent it from carrying out the Project in accordance with this Agreement and its annexes, including without limitation conflicts of interest with its employees, public administrations, with the national health service, with other Agreements, contracts and/or arrangements, or with applicable regulations. In the event that a conflict arises during the Term**,** the Foundation shall notify BMS of this fact, and shall comply with BMS's reasonable instructions to resolve the conflict (which may include termination of this Agreement).

TWELFTH - TRANSPARENCY

Notwithstanding the provisions of Clause Seven, the parties agree that BMS may disclose and/or publicly disseminate in appropriate forums and media (including websites) the terms of this Agreement, the activities relating to the Project and the fees and expenses associated with this Agreement for transparency purposes in accordance with applicable law and the Pharmaceutical Industry Code of Practice.

The parties undertake to cooperate with BMS to comply with the transparency commitments to which it is subject and, if necessary, to submit such information as BMS may reasonably require in order to comply with those commitments in relation to the publication of transfers of value.

THIRTEENTH - ASSIGNMENT OF THE AGREEMENT

Neither party may assign its rights and obligations under this Agreement or transfer or novate this Agreement without the prior written consent of the other party, which may not be unreasonably withheld or delayed.

FOURTEENTH - AMENDMENTS TO THE AGREEMENT

Any modification occurring after the signature of the Agreement shall be made in writing, and with the prior agreement of both parties, by means of the subscription of the corresponding addendum.

If for any reason any provision of this Agreement is declared invalid, this shall not affect the validity of the remainder of the Agreement and the terms of the Agreement shall remain in full force and effect, provided that such provision is independent of the remainder and is not of such importance that without it the Agreement would not have been concluded.

FIFTEENTH - NOTIFICATIONS

Notices to be given by the parties under this Collaboration Agreement shall be in writing (including by e-mail), and shall be deemed to have been received when delivered in person, or by electronic means with proof of delivery, or when five working days have elapsed since the date of dispatch, in cases where they have been sent by registered post. Notices shall be sent to the addressees below, unless updated by the parties by written notice (including by e-mail):

- On the part of BMS:
    - Contact person: Patricia de la Concha
    - Postal address: Calle Quintadueñas 6, 28050 de Madrid
    - E-mail: Patricia.delaConcha@bms.com
- On the part of the Foundation
    - Contact person: Carlos Moreno Dorado
    - Postal address: Instituto de Investigación Biomédica de Salamanca (IBSAL). Complejo Asistencial Universitario de Salamanca. Hospital Virgen de la Vega, 10th floor. Paseo de San Vicente, 58-182. CP 37007
    - E-mail: asesoria.juridica@ibsal.es
- By the Project Manager:

- Contact person: Dr. Emilio Fonseca Sánchez
- Postal address: Complejo Asistencial Universitario de Salamanca, Servicio de Oncología. P.º de San Vicente, 58-182, 37007 Salamanca
- E-mail: efonseca@usal.es; efonseca@saludcastillayleon.es

SIXTEENTH - PHARMACOVIGILANCE

i. The Foundation and/or the Project Manager shall immediately notify BMS of all "Adverse Events" and "Other Notifiable Events" associated with a BMS product no later than one (1) business day or three (3) calendar days, whichever occurs first, after becoming aware of the event. The definition of "Adverse Events" and "Other Notifiable Events" are defined at http://www.globalbmsmedinfo.com/.

ii. The Foundation and/or the Project Leader shall use the specific notification form provided by BMS and shall submit all information reasonably requested by BMS.

iii. Notifications should be sent in English by email to worldwide.safety@bms.com, by phone at +1.609.818.3804 or by telephone at +1.609.818.3804.
+1.609.818.3737.

iv. Case-by-case reconciliation.

1. To the extent that the Service involves the collection of primary data, The Foundation and/or the Project Manager will carry out a case-by-case reconciliation as instructed by BMS to confirm that BMS has received all reports from the Foundation and/or the Project Manager. Primary data collection means the collection of data directly from an individual, originally collected for the purposes of the project. The Foundation and/or Project Officer will initiate reconciliation activity by emailing AEPBUSINESSPROCESS@BMS.com to request a reconciliation report.

2. Reconciliation shall take place every three months, unless otherwise agreed by BMS in writing.

v. Training. All Foundation personnel and/or the Project Manager shall complete training reasonably required by BMS on security information, and shall certify or attest to the completion of such training, as reasonably requested by BMS. To complete adverse event training, please visit http://bmsmrt.pharmacertify.com.

vi. The Foundation and/or the Project Leader shall carry out its security obligations in compliance with the applicable regulations, including those relating to the protection of personal data.

vii. The Foundation and/or the Project Leader shall comply with reasonable additional information requests from BMS, if necessary.

viii. The Foundation and/or the Project Leader shall promptly notify BMS of "Product Quality Claims" (as defined at http://www.globalbmsmedinfo.com) associated with a BMS product no later than one (1) business day or three (3) calendar days after becoming aware of the claim. For notification purposes, the Foundation and/or the Project Leader shall send all available information about the complaint to the BMS contact in the country where the notifier is located. The BMS contact details can be found on the website http://www.globalbmsmedinfo.com. In the event that the BMS contact information for the country where the notifier is located is not available or is not provided, the Foundation and/or the Project Officer should notify the BMS contact for the United States. The Foundation and/or the Project Leader shall comply with reasonable follow-up requests from BMS on reported "Product Quality Claims".

SEVENTEENTH.- MISCELLANEOUS

1. The parties expressly acknowledge that under no circumstances shall BMS provide, on the basis of this Agreement, direct funding of any kind for the development of activities by the Foundation other than those expressly agreed in this Agreement.

2. The parties are independent contractors and nothing in this Agreement shall imply a partnership, agency or joint venture relationship between the parties or between the parties and their respective personnel. In this regard, in no event shall the personnel of any party be deemed to be the personnel of any other party. Each party is responsible for complying with all applicable labour, tax and other obligations. Neither party shall be liable for any statement, act or omission of the other party that is contrary to the provisions of this Clause.

3. This Agreement contains the entire agreement between the parties on the same subject matter and supersedes and replaces any previous agreement, oral or written, reached by the parties.

4. Nothing in this Agreement shall be deemed to constitute an identity of parties, or that one party shall be deemed to be the agent of the other. Neither party shall be liable for any statement, act or omission of the other party contrary to the foregoing.

5. The failure of either party to enforce any of its rights under this Agreement shall not be deemed to constitute a waiver of such rights in the future.

6. The Foundation and the Project Leader shall comply with reasonable monitoring requests by the Company and provide such information as the Company may reasonably request in relation to the Project.

7. This Agreement shall be governed by and construed in accordance with Spanish law. The parties agree

undertake to try to resolve amicably through the Monitoring Committee any differences that may arise in relation to this Agreement. In the event that it is not possible to reach an amicable solution regarding the interpretation and application of this Agreement, the Courts and Tribunals of Madrid (capital city) shall have jurisdiction, and the parties expressly waive any other jurisdiction that may correspond to them.

And in witness whereof, they have signed this Agreement in triplicate at the place indicated in the heading. The electronic signature of this Agreement shall have the same legal effect, validity and enforceability as a handwritten signature, and shall take full effect as of the last date of signature.

Bristol-Myers Squibb S.A.U.

Roberto Urbez
Roberto Urbez (Dec 5, 2022 15:43 GMT+1)

s: Roberto Úrbez

General Manager, Spain & Portugal

Date: Dec 5, 2022

The Foundation

Maria de Lorenzo
Maria de Lorenzo (Dec 12, 2022 14:46 GMT+1)

S.D.: María de Lorenzo Santiago

Director of Management of IBSAL

Date: Dec 12, 2022

Responsible for the project, as known and in agreement: Dr. Emilio Fonseca Sánchez

Signature: *EMILIO FONSECA SÁNCHEZ*
EMILIO FONSECA SÁNCHEZ (Dec 5, 2022 19:08 GMT+1)

Date: Dec 5, 2022

ANNEX I

COLLABORATIVE ACTIVITIES

1. Description of the project and a i m s of the collaboration

The aim of the PASOS Project is to collaborate with hospitals to carry out a Functional Rehabilitation Programme for oncology patients with associated respiratory pathology, in order to improve their respiratory capacity.

To this end, with the help of the Foundation, the figure of the Coordinator will be implemented in the Hospital's oncology service, with a dual function:

1. Carry out the Functional Rehabilitation Programme for oncology patients selected by the oncologist.
2. Work towards improving the project's efficiency indicators and measure and report on them on a n ongoing basis.

The oncologist will be responsible for selecting the patients likely to benefit from this project. However, in no case will such patient data be provided to BMS or to the supplier contracted by BMS for the preparation of the final report, if required.

During the first phase of the Project, the Project Manager, together with the help of BMS, will be responsible for defining the Functional Rehabilitation Programme and translating it into a document, as well as defining the materials for the continuation of the Programme at the patient's home.

Therefore, the Project Manager, with the support of BMS, will draw up a model action guide including:

- Definition of the Functional Rehabilitation Programme.
- Description of the roles of the entire team of health professionals involved.
- Description of the variables to measure the results of the effectiveness of this new care process model.
- Supporting materials, if needed: project information card, patient materials for monitoring the programme at home, and excel for monitoring indicators.

BMS will provide whatever assistance is necessary for the success of such implementation.
BMS will make a contribution to the Foundation to cover the expenses derived from the implementation of the project. This amount is the maximum contribution to be made by BMS for the management of the project at the Hospital, and will be paid in a single payment upon signing the collaboration agreement.

2. Project duration and implementation schedule

The project will have a duration of 12 months, extendable by agreement between the Parties. The phases of the project are:

I. Definition of the Functional Rehabilitation Programme and patient materials. In this phase the measurement indicators of the project will also be defined.
II. Project implementation.
III. Measurement of results according to indicators defined in Phase I.

3. Measuring Results

In order to demonstrate the benefits of this project, a number of success indicators related to the key objectives of the project will be measured:

- Number of oncology patients with associated respiratory pathology in the hospital
- Number of patients included in the project
- Reduced length of stay in hospital
- Decrease in readmissions
- Improving Quality of Life: SF-12 questionnaire
- Decrease in caregiver strain: measured by the ZARIT questionnaire.
- Patient satisfaction, measured by means of a specific liekert-type patient satisfaction survey, prepared for the implementation of the project.

In order to analyse the results of the Project, BMS may contract a supplier who will analyse the anonymised data, where appropriate, collected by the Foundation and the Project Manager and will draw up a final report with the conclusions on the established indicators that will allow the impact of the Project to be assessed.

ANNEX II
BREAKDOWN OF THE FINANCIAL CONTRIBUTION

BMS's financial contribution within the framework of this agreement will be used to cover part of the expenses, both human and material, derived from the management and follow-up of oncology patients with respiratory pathology, selected by the clinician, among them:

- Programme design, implementation and patient follow up
- Materials for monitoring the home-based programme
- Indirect project costs.

BMS shall pay the amount of 15.000 € within sixty (60) days of the presentation to BMS of the corresponding letter of payment to the bank account: IBAN ES31 2100 8690 8702 0000 9719 that the Foundation has opened with CAIXABANK. BMS will pay the applicable taxes and make the legally required withholdings.

Any modification or extension of the amount of the collaboration shall require the express prior written agreement of the parties.

The Entity undertakes to allocate the amount of the collaboration to the implementation of the Project.

CW3815708 - CW3815708_Convention PASOS project collaboration vf.docx

Final Audit Report 2022-12-12

Created:	2022-12-05
By:	valeria franco (valeria.franco@bms.com)
Status:	Signed
Transaction ID:	CBJCHBCAABAAPcDSuj34gZAjrtpa72G_TLl2p3a4AhJq

"CW3815708 - CW3815708_Convenio Colaboración proyecto PASOS vf.docx" History

Document created by valeria franco (valeria.franco@bms.com)
2022-12-05 - 12:36:38 PM GMT

Document emailed to roberto.urbez@bms.com for signature
2022-12-05 - 12:37:37 PM GMT

Document emailed to Maria de Lorenzo (dir.gestion@ibsal.es) for signature
2022-12-05 - 12:37:37 PM GMT

Document emailed to EMILIO FONSECA SÁNCHEZ (efonseca@usal.es) for signature
2022-12-05 - 12:37:38 PM GMT

Email viewed by EMILIO FONSECA SÁNCHEZ (efonseca@usal.es)
2022-12-05 - 2:06:02 PM GMT

Email viewed by roberto.urbez@bms.com
2022-12-05 - 2:43:12 PM GMT

Signer roberto.urbez@bms.com entered name at signing as Roberto Urbez
2022-12-05 - 2:43:32 PM GMT

Document e-signed by Roberto Urbez (roberto.urbez@bms.com)
Signature Date: 2022-12-05 - 2:43:34 PM GMT - Time Source: server

Document e-signed by EMILIO FONSECA SÁNCHEZ (efonseca@usal.es)
Signature Date: 2022-12-05 - 6:08:25 PM GMT - Time Source: server

Email viewed by Maria de Lorenzo (dir.gestion@ibsal.es)
2022-12-12 - 7:51:21 AM GMT

New document URL requested by Maria de Lorenzo (dir.gestion@ibsal.es)

2022-12-12 - 1:44:33 PM GMT

Document e-signed by Maria de Lorenzo (dir.gestion@ibsal.es)

Signature Date: 2022-12-12 - 1:46:12 PM GMT - Time Source: server

Agreement completed.

2022-12-12 - 1:46:12 PM GMT

VNiVERSiDAD
D SALAMANCA
CAMPUS DE EXCELENCIA INTERNACIONAL

SOLICITUD DE AUTORIZACIÓN PARA CONTRATOS AL AMPARO DEL ART. 83 DE LA LEY ORGÁNICA DE UNIVERSIDADES

1. DATOS DEL SOLICITANTE

1.1. Primer apellido	1.2. Segundo apellido	1.3. Nombre
**Fernández**	**Rodríguez**	**Eduardo José**
1.4. Reg. personal	1.5. Categoría académica<br>**Profesor Ayudante Doctor**	1.6. Área de conocimiento<br>**Fisioterapia**
1.7. Departamento/Instituto/Centro/Grupo/Unidad<br>**Departamento Enfermería y Fisioterapia**		

2. PERSONA O ENTIDAD CON LA QUE DESEA CONTRATAR:

2.1. Denominación	2.2. Tipo de actividad
**INSTITUTO DE INVESTIGACIÓN BIOMÉDICA DE SALAMANCA (IBSAL) DE LA FUNDACIÓN INSTITUTO CIENCIAS DE LA SALUD DE CASTILLA Y LEÓN**	**Prestación de Servicios Técnicos Sanitarios**
2.3. Domicilio y localidad<br>**Paseo San Vicente nº 58-182**	2.4. CIF/NIF<br>**G37576360**

3. CONTENIDO DEL CONTRATO

3.1. Título y/o resumen	
Programa de reeducación en actividades de la vida diaria y prescripción de productos de apoyo en el paciente oncológico. Seguimiento del paciente oncológico desde la perspectiva de la terapia ocupacional.	
3.2. Duración<br>**12 meses**	3.3. Importe económico<br>**13.950,00**

4. P.D.I. QUE SE COMPROMETE A ASUMIR LA EJECUCIÓN DEL CONTRATO:

4.1. Nombre y apellidos	4.2. Categoría académica	4.3. Horas semanales estimadas de dedicación	FIRMA DEL PROFESOR
**Eduardo J Fernández Rodríguez**	**Profesor Ayudante Doctor**	**4**	FERNANDEZ RODRIGUEZ EDUARDO JOSE - 70863702N Firmado digitalmente por FERNANDEZ RODRIGUEZ EDUARDO JOSE - 70863702N Fecha: 2023.01.19 10:53:50 +01'00'

Firma: FERNANDEZ RODRIGUEZ EDUARDO JOSE - 70863702N Firmado digitalmente por FERNANDEZ RODRIGUEZ EDUARDO JOSE - 70863702N Fecha: 2023.01.19 10:53:07 +01'00'

Fdo.: **D./Dña. Eduardo José Fernández Rodrígez**

Salamanca a **19** de **enero** de **2023**

El/la **CONSEJO DE DEPARTAMENTO DE ENFERMERIA Y FISIOTERAPIA** acordó con fecha **20** de **enero** de **2023** informar favorablemente la realización del citado contrato.

Firma: UNIVERSIDAD DE SALAMANCA - Departamento de Enfermería y Fisioterapia

Firmado digitalmente por MARTIN NOGUERAS ANA MARIA - 07959908E
Nombre de reconocimiento (DN): c=ES, serialNumber=IDCES-07959908E, givenName=ANA MARIA, sn=MARTIN NOGUERAS, cn=MARTIN NOGUERAS ANA MARIA - 07959908E
Fecha: 2023.01.20 13:03:35 +01'00'

V.º B.º y CONFORME
El responsable de
Departamento/Instituto/Centro/Grupo/Unidad

PRESUPUESTO ECONÓMICO DEL CONTRATO

5. INGRESOS

5.1. Importe del contrato			13.950,00
5.2. I.V.A.	**0%**		0
		**5.3. INGRESOS TOTALES**	13.950,00

6. GASTOS MATERIALES Y PERSONALES DE EJECUCIÓN

6.1. Material inventariable				
6.2. Viajes y dietas				
6.3. Material fungible				
6.4. Servicios profesionales externos				
6.5. Becarios				
6.6. Personal contratado				
6.7.				
6.8. P.A.S. (Relación nominal, retribución + SS)				
6.9. Gastos generales de la Universidad	**12%**	del importe del contrato (5.1)		1.674,00
6.9.1. Dpto./Centro/Grupo		(20% de 6.9.)	334,80	
6.9.2. Universidad de Salamanca		(20% de 6.9.)	334,80	
6.9.3. Vicerrectorado de Investigación		(60% de 6.9.)	1004,40	
		**6.10. GASTOS TOTALES**		1.674,00
		**DIFERENCIA (5.1 - 6.10)**		12.276,00

7. RETRIBUCIÓN DEL P.D.I. **12.276,00**

Nombre del profesor	N.I.F.	
**Eduardo José Fernández Rodríguez**	**70863702N**	12.276,00

8. DPTO/INSTITUTO/CENTRO/GRUPO/UNIDAD **0,00**

(Adicional al importe 6.9.1)

Nombre

Salamanca a **21** de **diciembre** de **2022**

Firma:

FERNANDEZ RODRIGUEZ EDUARDO JOSE - 70863702N

Firmado digitalmente por FERNANDEZ RODRIGUEZ EDUARDO JOSE - 70863702N
Fecha: 2023.01.20 13:58:43 +01'00'

Fdo.: **D./Dña.** **Eduardo José Fernández Rodríguez**

HOJA DE ENCARGO DE PRESTACIÓN DE SERVICIOS (Art.83 L.O.U.) CON LA UNIVERSIDAD DE SALAMANCA (1)

DATOS DE LA ENTIDAD, EMPRESA O PERSONA FÍSICA SOLICITANTE		
NOMBRE: **INSTITUTO DE INVESTIGACIÓN BIOMÉDICA DE SALAMANCA (IBSAL) DE LA FUNDACIÓN INSTITUTO CIENCIAS DE LA SALUD DE CASTILLA Y LEÓN**		C.I.F. **G37576360**
DOMICILIO: **Paseo San Vicente nº 58-182**		
LOCALIDAD: **SALAMANCA**	Código Postal: **37007**	TELÉFONO: 923 09 04 70
REPRESENTADA POR D/Dña.: María de Lorenzo Santiago		
DNI: 07.969.495-H	CARGO: Directora de Gestión del IBSAL	e-mail: dir.gestion@ibsal.es

DATOS DE LA UNIVERSIDAD DE SALAMANCA
PROFESOR RESPONSABLE: EDUARDO JOSÉ FERNÁNDEZ RODRÍGUEZ
DEPARTAMENTO, INSTITUTO O CENTRO: ENFERMERÍA Y FISIOTERAPIA

DATOS DEL SERVICIO SOLICITADO		
DESCRIPCIÓN DEL SERVICIO (2):<br>Programa de reeducación en actividades de la vida diaria y prescripción de productos de apoyo en el paciente oncológico. Seguimiento del paciente oncológico desde la perspectiva de la terapia ocupacional.		
FECHA DE INICIO: 15/01/2023	IMPORTE:	13950
FECHA PREVISTA DE FINALIZACIÓN:	IVA (-) (3):	-
	TOTAL):	13950

OBSERVACIONES: El I.V.A. no es aplicable ya que se trata de una actividad sanitaria.

En Salamanca, a 13 de enero de 2023

07969495H MARÍA DE LORENZO (R: G37576360) — Firmado digitalmente por 07969495H MARÍA DE LORENZO (R: G37576360) Fecha: 2023.01.13 14:43:31 +01'00'

Por la Empresa

FERNANDEZ RODRIGUEZ EDUARDO JOSE - 70863702N — Firmado digitalmente por FERNANDEZ RODRIGUEZ EDUARDO JOSE - 70863702N Fecha: 2023.01.13 10:34:50 +01'00'

Por la Universidad de Salamanca
(Profesor responsable)

(1) Por favor, una vez cumplimentado este impreso, y debidamente **firmado y sellado**, remítalo a la OTRI de la Universidad de Salamanca (USAL). Edificio I+D+i. C/ Espejo, s/n.37007-Salamanca
(2) El investigador responsable deberá entregar a la Empresa por medio fehaciente cada uno de los informes parciales o final, o cualquier tipo de entregable derivado de los trabajos del personal de la USAL, y la Empresa deberá acusar recibo de entrega, disponiendo de diez días naturales desde la fecha de recepción para manifestar cualquier disconformidad con el contenido de los mismos. La no emisión de disconformidad supondrá la aceptación del entregable.
(3) Si el I.V.A. no es aplicable o difiere del 21%, por favor consígnenlo en el apartado de Observaciones.
